# Alpine Grassland Growth and Its Ecological Responses to Environmental Impacts: Insights from a Comprehensive Growth Index and SHAP-Based Analysis

**DOI:** 10.3390/plants15010093

**Published:** 2025-12-27

**Authors:** Yanying Li, Yongmei Liu, Xiaoyu Li, Junjuan Yan, Yuxin Du, Ying Meng, Jianhong Liu

**Affiliations:** 1 College of Urban and Environmental Sciences, Northwest University, Xi’an 710127, China; liyanying@stumail.nwu.edu.cn (Y.L.); 202221149@stumail.nwu.edu.cn (X.L.); 13581391665@163.com (J.Y.); 202421691@stumail.nwu.edu.cn (Y.D.); nimo@stumail.nwu.edu.cn (Y.M.); jhliu_85@163.com (J.L.); 2 Shaanxi Key Laboratory of Earth Surface System and Environmental Carrying Capacity, Xi’an 710127, China

**Keywords:** comprehensive growth index, vegetation index, SHAP-enhanced machine learning, alpine grassland, Qinghai Province

## Abstract

The alpine grassland is one of the most representative ecosystems on the Qinghai–Tibet Plateau, Growth monitoring is fundamental for the alpine grassland maintenance and husbandry sustainability. In this study, by the integration of regression model, principal component analysis, and SHAP-enhanced machine learning, a comprehensive growth index (CGI) was proposed for the accurate and quick assessment of alpine grassland growth in Qinghai Province, located in the eastern Qinghai–Tibet Plateau. The temporal and spatial growth behaviors of the main grassland types over 2001–2023 were then determined and the differences in key driving factors and their responses explored. The results indicated that the CGI composed of KNDVI, EVI, MSAVI, GNDVI and CVI characterized the typical ecological and physical parameters related to grassland growth, proved to be optimal and efficient in long-term growth monitoring. Alpine grassland growth fluctuated but gradually increased from 2001 to 2023, but individual types exhibited different trends. In particular, the two main types of alpine meadow and alpine steppe displayed the weakest increasing trend in growth, with the good-growth and continuous-increasing area proportions of 26.01% and 18.03%, 70.45% and 74.72%, respectively. Soil total nitrogen was the most critical common factor and significantly increased the growth across all five grassland types, then followed by grazing intensity and precipitation, which exhibits diverse effects on the individual types. The result implies the significant heterogeneity in the key driviers which affect the alpine grassland growth over large scale.

## 1. Introduction

Grasslands are essential components of terrestrial ecosystems, contributing to bio-diversity conservation, climate regulation, soil and water conservation, and carbon se-questration, while also supporting livestock production [1,2]. The growth of grassland vegetation is highly sensitive to climate variability and strongly influenced by anthro-pogenic activities [3,4]. Therefore, accurate monitoring of grassland growth is critical for ecosystem maintenance, grassland management, and husbandry sustainability.

Grassland growth refers to the growth status and variation of grassland vegetation [5]. Numerous studies have demonstrated strong and stable relationships between in situ grassland observations and remotely sensed vegetation metrics. Key vegetation ecological parameters, such as aboveground biomass (AGB) and fractional vegetation cover (FVC), are widely used to assess vegetation growth and correlate strongly with spectral indices derived from remote sensing [6,7]. Satellite-based monitoring offers significant advantages for long-term, large-scale vegetation assessment [8]. Vegetation indices (VIs) derived from multispectral reflectance, such as normalized difference vegetation index (NDVI), kernel normalized difference vegetation index (kNDVI), and enhanced vegetation index (EVI), are widely used to represent canopy biomass, chlorophyll content, and nitrogen status [9,10]. Other vegetation indices, including the ratio vegetation index (RVI), modified soil-adjusted vegetation index (MSAVI), green chlorophyll index (GCI), chlorophyll vegetation index (CVI), green normalized difference vegetation index (GNDVI), and Normalized difference vegetation index green-blue (NDVI_green–blue_) are sensitive to diverse biophysical traits, thus providing specific vegetation information [11,12,13,14,15,16,17,18]. Composite indices, such as the growth index, which combines NDVI and EVI [19], and the composite vegetation index, which integrates NDVI, EVI, near-infrared reflectance vegetation index (NIRv), and kNDVI [20], have shown improved performance in the growth monitoring of cropland and arid ecosystems. Therefore, integrating multiple vegetation indices, rather than relying on a single index, may offer a more accurate representation of alpine grassland dynamics [20]. However, the applicability of such comprehensive indices remains insufficiently evaluated.

Climatic, topographic, soil, and anthropogenic factors jointly influence grassland dynamics [21,22,23,24], yet vegetation-type-specific differences in their relative environmental impacts remain poorly understood [25,26]. Traditional methods such as linear regression, residual analysis, and GeoDetector have limited ability to capture nonlinear interactions [27,28]. Machine learning models, such as random forest (RF), boosted regression trees (BRT), and extreme gradient boosting (XGBoost), have been increasingly applied in ecological studies with improved accuracy and robustness [29,30,31]. The SHapley Additive exPlanations (SHAP) method further enhances interpretability by quantifying variable contributions, offering an effective framework for identifying heterogeneous vegetation responses [32]. Consequently, the interpretable machine learning models integrated with SHAP provide an effective solution for addressing vegetation-type-specific environmental controls.

Environmental heterogeneity affects plant biophysical characteristics, reflecting complex and multifactor controls. One study reported that leaf traits in Chinese grasslands exhibit stronger soil–trait coupling in narrow-ranging species than in wide-ranging ones [33]. Another study indicated that morphological variability may primarily reflect ecological and genetic differentiation rather than direct environmental forcing [34]. Remote sensing has also proven effective for capturing vegetation development across heterogeneous landscapes, through the fusion of Landsat and MODIS imagery for monitoring crop growth [35]. These findings highlight the importance of accounting for vegetation-type-specific differences when developing remote sensing approaches for evaluating alpine grassland growth.

Qinghai Province, located in the northeastern Qinghai–Tibetan Plateau, contains widespread alpine meadows and alpine steppes that are highly sensitive to climate change and human disturbance. Alpine meadows are dominated by cold-tolerant perennial herbs with dense canopies, whereas alpine steppes are characterized by xerophytic grasses with sparser structures [36]. Different degrees of grassland degradation have been reported in recent decades [37,38]. Therefore, this study integrates MODIS vegetation products with climatic, topographic, soil, and anthropogenic datasets, with the objectives of (1) constructing a comprehensive growth index (CGI) model for major alpine grassland types and analyzing its spatiotemporal variation during 2001–2023, and (2) identifying key environmental drivers and vegetation-type-specific response mechanisms using interpretable machine learning.

## 2. Materials and Methods

### 2.1. Study Area

**Figure 1:**
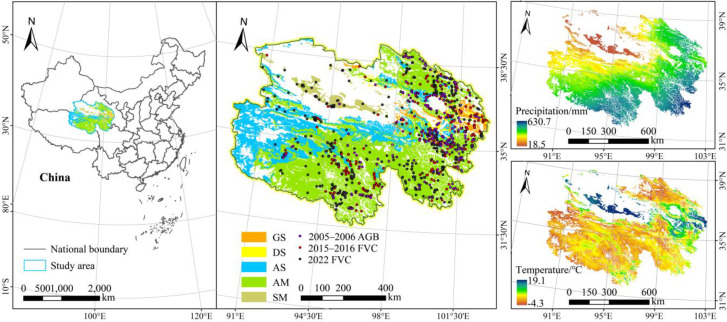
Study area. GS: gramineous steppe; DS: desert steppe; AS: Alpine steppe; AM: Alpine meadow; SM: saline meadow.

Qinghai Province is located in western China (31°36′–39°12′ N, 89°24′–103°04′ E), covering approximately 7.22 × 10^5^ km^2^ with a mean elevation exceeding 3000 m. The region is characterized by plateau landforms, featuring higher elevations in the west and lower elevations in the east, as well as lower terrain in the central area relative to the north and south. It has a typical plateau continental climate, with mean annual temperatures ranging from −6 to 9 °C and annual precipitation of 250–550 mm. Since the mid-20th century, the northeastern Qinghai–Tibet Plateau (including Qinghai Province) has exhibited a pronounced warming–wetting trend, reflected in long-term increases in temperature and precipitation; however, frequent extreme drought events persist in certain regions [39,40]. Grasslands occupy approximately 4.19 × 10^5^ km^2^ of the province [41] and are mainly classified into alpine meadow, alpine steppe, gramineous steppe, desert steppe, and saline meadow. The alpine meadow is the dominant type (63.87%), followed by the alpine steppe (25.90%); both types are primarily distributed in the Qilian Mountains, the Qinghai Lake Basin, and the Three-River Source Region. The remaining grassland types each account for less than 5% of the total area and are mainly distributed in the Huangshui Basin, the Qinghai Lake Basin, the Qaidam Basin, and the Gonghe Basin (Figure 1).

### 2.2. Data Collection

#### 2.2.1. Remote Sensing Data

Although earlier satellite records are available since the 1980s, their coarse spatial resolution and spectral inconsistencies limit their comparability with MODIS products. To ensure the consistency of vegetation indices, MODIS MOD13Q1 and MOD09A1 datasets for the period May–September 2001–2023 were used as the primary data sources for vegetation index calculations, and the detailed parameters are listed in Table 1. Using the Google Earth Engine (GEE) platform (Google LLC, Mountain View, CA, USA; https://earthengine.google.com, accessed on 26 July 2025), the quality assessment (QA) band was employed for noise filtering, linear interpolation was applied to fill data gaps, and the time series data were subsequently smoothed using the Savitzky–Golay filter [42]. The maximum value composite (MVC) was generated monthly from the processed data, and the growing-season means were calculated for May–September. All the datasets were consistently reprojected to the WGS_1984_Albers coordinate system, clipped to the Qinghai Province boundary, and resampled at a 250 m spatial resolution.

#### 2.2.2. Vegetation Data

Field AGB and FVC data were used to select the optimal combination of vegetation indices and construct the CGI models. The AGB dataset was obtained from 1495 field sampling sites during July–August 2005–2006 in Qinghai Province and was provided by Xia et al. [43]. Sampling sites were selected based on community representativeness; each site covered an area of at least 100 m × 100 m, and 3–5 quadrats (1 m × 1 m) were established within each site for AGB measurement. The FVC dataset was provided by the Qinghai Grassland Supervision Center [44], which included 903 ground-survey quadrats collected during July–August 2015–2016, with herbaceous quadrats measuring 1 m × 1 m.

The mean ± 3 standard deviations criterion was used to eliminate outliers. When two or more quadrats were located within the same 250 m pixel, their average value was taken as the sample value. The number and spatial distribution of the measured AGB and FVC quadrats are shown in Table 2 and Figure 1.

FVC data derived from Google Earth images from July to August 2022 was used to validate the constructed CGI models. A total of 376 samples were deployed in Qinghai Province based on the typicality of grassland types. The blue, green, and red band reflectance values were used to calculate the excess green index (EXG), which was subsequently applied to derive grassland vegetation cover. Each sample covered an area of 250 m × 250 m, which is consistent with the resolution of the MODIS image, and the sample distribution is presented in Figure 1.

Alpine grassland categories were obtained from the Vegetation Map of the People’s Republic of China (1:1,000,000) [45], specifically the section covering Qinghai Province, which was downloaded from the Plant Science Data Center of the Chinese Academy of Sciences (https://www.plantplus.cn/cn).

#### 2.2.3. Environmental Datasets

Climatic, topographic, soil, and anthropogenic factors were derived using relevant environmental datasets (Table 3). Climatic factors included the growing-season total monthly precipitation (PRE) and the mean monthly values of temperature (TMP), potential evapotranspiration (PET), downward surface shortwave radiation (DSSR), and the palmer drought severity index (PDSI). Topographic factors, including slope and aspect, were derived from the 30 m digital elevation model (DEM). Soil factors included the mean values of total soil nitrogen (STN), total soil phosphorus (STP), total soil potassium (STK), soil PH (SPH), and soil water (SW) within the 0–100 cm soil layer. Anthropogenic activity factors included grazing intensity (GI) and population (POP). Climatic and anthropogenic data spanned the period 2001–2023, and all data were reprojected to the WGS_1984_Albers coordinate system and resampled to 250 m resolution.

### 2.3. Methodology

**Figure 2:**
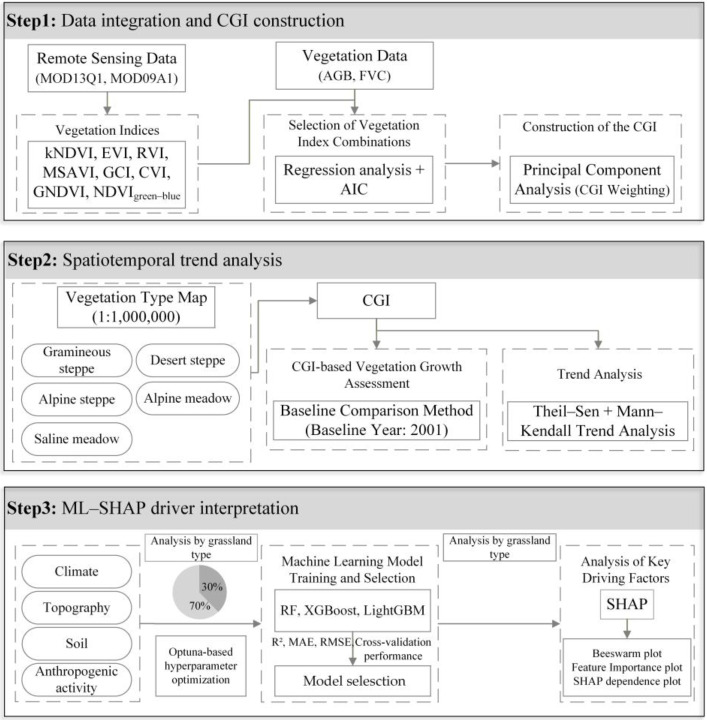
Research framework.

The research framework (Figure 2) presents the workflow adopted in this study, which integrates vegetation index derivation from MODIS products, CGI construction, spatiotemporal analysis, and SHAP-enhanced interpretation of environmental drivers. Eight vegetation indices were first derived from the MOD13Q1 and MOD09A1 datasets using the GEE platform, and the optimal index combination was identified through regression analysis and the Akaike Information Criterion (AIC). A comprehensive growth index (CGI) was then constructed by assigning weights using principal component analysis (PCA). Baseline comparison and trend analysis were applied to quantify the spatiotemporal differentiation of alpine grassland growth across Qinghai Province. Using the CGI as the response variable and climatic, topographic, soil, and anthropogenic factors as predictors, three machine-learning models (Random Forest, XGBoost, and LightGBM) were implemented. Finally, the SHAP method was employed to interpret the relative contributions and nonlinear effects of key environmental drivers across different alpine grassland types.

#### 2.3.1. Vegetation Indices Calculation

Vegetation indices are closely related to vegetation biophysical parameters and serve as key indicators for monitoring vegetation growth status and dynamics. Therefore, eight vegetation indices were derived from MODIS data using the GEE platform, with EVI directly obtained from the MOD13Q1 product. These indices collectively capture multiple aspects of vegetation growth, including canopy structure, vegetation cover, biomass, chlorophyll-related properties, and nitrogen-related information (Table 4). They are ecologically significant and comprehensive, thus selected as candidate indices.

#### 2.3.2. Regression Analysis

In this study, optimal vegetation index combinations were identified by performing regression analyses on measured FVC and AGB quadrat data from 2005, 2006, 2015, and 2016, considering all possible combinations of eight vegetation indices, with at least two indices per combination. Using the least squares method, four statistical metrics were calculated for the linear regression models relating FVC and AGB to each vegetation index combination: the adjusted coefficient of determination ($R_{\mathrm{adj}}^{2}$), root mean square error (RMSE), mean absolute error (MAE), and AIC [48]. These metrics were used to evaluate model accuracy. $R_{\mathrm{adj}}^{2}$ mitigates the issue of inflated coefficient of determination (R^2^) values in regression models that arise from increasing the number of vegetation indices in the combination. Lower RMSE and MAE values indicate better model predictive performance. AIC is used to identify the optimal model by balancing goodness of fit and model complexity, with smaller values indicating a better trade-off [49] (Formulas (1)–(5)).

Each of the four metrics was normalized, and negative indicators were converted to positive values using the (1 − x) transformation. The sum of these normalized values represented the composite score for each year’s vegetation index combination (score_j_), which reflects the regression model’s accuracy between the measured FVC and AGB. Finally, the composite scores from all four years were summed to obtain the overall accuracy score (total score). A higher value indicates greater predictive accuracy of the vegetation index combination for FVC and AGB, signifying a more precise characterization of grassland vegetation growth.

(1) $$R_{\mathrm{adj}}^{2}=1-\left(1-R^{2}\right)\frac{n-1}{n-k-1}$$

(2) $$R^{2}=1-\frac{\sum_{i=1}^{n}{\left(y_{i}-{\hat{y}}_{i}\right)}^{2}}{\sum_{i=1}^{n}{\left(y_{i}-\bar{y}\right)}^{2}}$$

(3) $$\mathrm{RMSE}=\sqrt{\frac{1}{n}\sum_{i=1}^{n}{\left(y_{i}-{\hat{y}}_{i}\right)}^{2}}$$

(4) $$\mathrm{MAE}=\frac{1}{n}\sum_{i=1}^{n}\;\left|y_{i}-{\hat{y}}_{i}\right|$$

(5) $$\mathrm{AIC}=\mathrm{nln}S_{p}^{2}+2[k+1]$$

(6) $${\mathrm{score}}_{j}=\sum_{i}^{m}{\tilde{V}}_{i,j}\left(m=4\right)$$

(7) $$\mathrm{total}\;s\mathrm{core}=\sum_{j\in \left\{2005,2006,2015,2016\right\}}{\mathrm{score}}_{j}$$

where n is the sample size, $S_{p}^{2}$ is the residual mean square, k is the number of variables in the model; ${\hat{y}}_{i}$ is the predicted value, $y_{i}$ is the actual value, and $\bar{y}$ is the mean value; ${\;\tilde{V}}_{i,j}$ denotes the normalized and directionally unified value of the i-th indicator in year j, score_j_ represents the comprehensive score in year j, and m is the number of accuracy evaluation indicators.

#### 2.3.3. Principal Component Analysis

To capture the responses of different vegetation indices to grassland vegetation growth parameters, a comprehensive growth index model was constructed using the selected optimal vegetation index combination, which served as an integrated indicator for grassland vegetation growth monitoring. PCA was employed to assign weights to each vegetation index within the model. This method provides an objective and efficient means of determining index weights on the basis of statistical variance rather than subjective judgment. Through principal component transformation, multiple correlated variables are converted into uncorrelated principal components. The loading coefficients of each original variable were multiplied by the variance contribution rate of each principal component, summed across all components, and then normalized to derive the final weight coefficients [50]. In the study, PCA was performed on the optimal combination of vegetation indices. The results revealed that the variance contribution rate of the first principal component (PC_1_) exceeded 98% throughout the 2001–2023 period. Therefore, PC_1_ was extracted to assign weights to each vegetation index.

The selected m vegetation indices were standardized to produce the index variables *x*_1_, *x*_2_, *…*, *x*ₘ. On the basis of PCA, PC_1_ was derived. Weights were calculated from the coefficients of each index variable in the linear combination of PC_1_; the CGI model was then constructed as shown in Equations (8)–(10).

(8) $$PC_{1}=a_{11}x_{1}+a_{12}x_{2}+\cdots +a_{1p}x_{m}$$

(9) $$w_{i}=\frac{a_{i}}{\sum_{i=1}^{m}\;a_{i}}$$

(10) $$\mathrm{CGI}\;=\sum_{i=1}^{m}w_{i}\times {\mathrm{VI}}_{i}$$

where a_1i_ denotes the loading of the i-th vegetation index in the linear combination of the PC_1_, that is, the element of the eigenvector corresponding to the largest eigenvalue; w_i_ is the weight of index x_i_; m is the total number of vegetation indices; and VI_i_ is the value of the i-th vegetation index.

#### 2.3.4. Vegetation Growth Analysis

In this study, the interannual variation characteristics of alpine grassland vegetation growth in Qinghai Province from 2001 to 2023 were analyzed on the basis of the annual mean CGI during the growing season. Moreover, the baseline comparison method was employed to calculate the differences in CGI between 2002 and 2023 and the baseline year 2001, thereby classifying grassland vegetation growth into different levels and examining the variation patterns of overall and type-specific grassland growth. The mean-standard deviation method was applied to classify the CGI difference values into five levels (Table 5), using μ ± 0.5 σ and ± 1.5 σ as the classification thresholds, where μ and σ denote the mean and standard deviation, respectively.

The Theil-Sen slope estimator (Sen’s slope) is a robust, nonparametric statistical method that is insensitive to measurement errors and outliers and is computationally efficient. The Mann–Kendall test (MK) does not require data to follow a normal distribution and is unaffected by missing values and outliers. The combination of these two methods has been widely applied to long-term vegetation trend analysis [51]. In this study, the CGI was used as the indicator of vegetation growth. Analysis was conducted on the GEE platform using a combination of Sen’s slope and Mann–Kendall (MK) methods to examine changes in vegetation growth across the alpine grasslands of Qinghai Province from 2001 to 2023. The results were classified into five categories (Table 6).

#### 2.3.5. Combination of Machine Learning Models and SHAP

In this study, interpretable machine learning models were employed to quantify the relative contributions of climate, topography, soil, and anthropogenic activities to the growth of different grassland vegetation types in Qinghai Province. A fishnet grid with a spatial resolution of 2 km × 2 km was constructed using ArcGIS Pro (v3.0.2; Esri Inc., Redlands, CA, USA). Based on this grid, sampling was performed on the CGI data for alpine grasslands and the corresponding thematic layers of influencing factors from 2001 to 2023. Invalid values were excluded to construct the dataset. The sample sizes for each vegetation type were as follows: 4638 for gramineous steppe, 1330 for desert steppe, 26,805 for alpine steppe, 65,565 for alpine meadow, and 4482 for saline meadow, totaling 102,820 samples. Regression models linking vegetation growth to environmental factors were constructed for different grassland types using RF, XGBoost, and LightGBM algorithms.

RF is an improvement over the classification and regression trees (CART) algorithm and determines the optimal splitting feature by randomly selecting feature subsets at each node. This randomness effectively reduces overfitting and increases model accuracy and robustness [52]. Compared with traditional linear statistical methods, XGBoost captures the nonlinear relationships between multiple predictors and the target variable. It offers advantages such as fast training speed and reduced susceptibility to overfitting [53]. Developed by Microsoft, LightGBM is an efficient implementation of gradient boosting decision trees (GBDT). It features improved training speed and memory efficiency and supports large-scale parallel computing [54].

The sample data were split into training and testing sets at a 7:3 ratio. Optuna was employed for hyperparameter optimization and combined with fivefold cross-validation to improve model generalizability. Specifically, the mean cross-validation error across folds was used as the optimization criterion, ensuring that model selection was guided by cross-validation performance rather than a single train–test split. Optuna transforms the hyperparameter optimization process into an optimization problem of black-box functions; it integrates Bayesian optimization with early-termination mechanisms to effectively reduce the computational costs associated with ineffective parameter combinations [55]. The key parameter, the number of decision trees (n_estimators), ranged from 50 to 3000 for gramineous steppe and saline meadow, from 50 to 500 for desert steppe, and from 50 to 5000 for alpine steppe and alpine meadow. The other model hyperparameter settings are detailed in Table 7. Models were built using scikit-learn (v1.6.1), XGBoost (v1.7.6), LightGBM (v4.6.0), and Optuna (v4.2.1) in a Python 3.11.11 environment, with R^2^, MAE, and RMSE used to evaluate model performance during cross-validation and on the independent testing set.

To overcome the limited interpretability of machine learning models, SHAP was used to quantify the contributions and interactions of each feature to the model’s output by calculating the Shapley value for each feature in every sample [32]. In this study, SHAP values were calculated for various influencing factors in the growth prediction models for different grassland vegetation types. The differential effects of environmental factors on the CGI of grassland vegetation growth in Qinghai Province were analyzed, and key influencing factors were identified. The aforementioned process primarily utilized the shap package (v0.46.0) in Python 3.11.11.

## 3. Results

### 3.1. CGI Model Construction

On the basis of regression analysis, the vegetation index combination with the highest fitting accuracy against the measured AGB and FVC was selected (Table 8). The results indicated that the combination of the KNDVI, EVI, MSAVI, GNDVI, and CVI most accurately characterized grassland vegetation parameters, with the highest total score of fitting accuracy (14.8057). Therefore, this combination was selected as the data source for constructing the CGI in this study.

**Figure 3:**
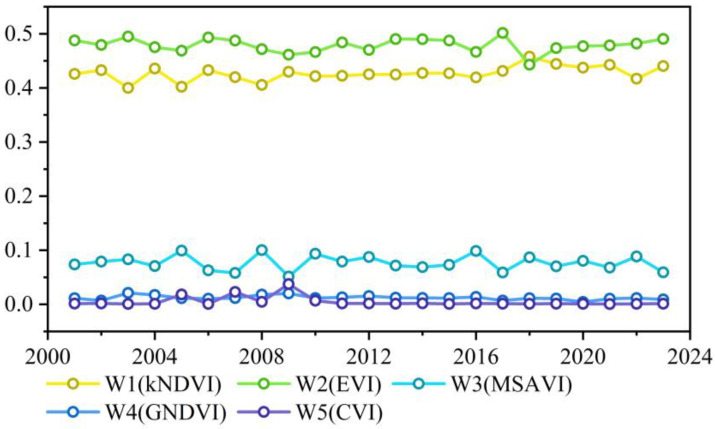
Weighting coefficients for vegetation indices in the CGI model (2001–2023).

Furthermore, on the basis of the optimal combination of vegetation indices (kNDVI, EVI, MSAVI, GNDVI, and CVI), PCA was applied to derive the weighting coefficients of each index to construct the CGI model for 2001–2023. The changes in the weights of each vegetation index from 2001 to 2023 were relatively gentle with certain fluctuations, as shown in Figure 3. The average weighting coefficients of the vegetation indices were ranked as follows: W2 (EVI, 0.4791) > W1 (kNDVI, 0.4270) > W3 (MSAVI, 0.0767) > W4 (GNDVI, 0.0123) > W5 (CVI, 0.0049). Among these indices, the kNDVI and EVI consistently dominated the CGI composition, with weighting coefficients significantly greater than those of the other three indices.

Linear regression analyses were subsequently conducted on FVC samples derived from 2022 Google Earth imagery against the corresponding CGI and KNDVI values for alpine grasslands in 2022 (Table 9). The results indicated that the regression accuracy of the CGI was higher than that of the kNDVI for FVC estimation, with an increase in R^2^ of 0.0189 and a decrease in the RMSE of 0.2468. This confirmed that the CGI achieved higher inversion accuracy in estimating FVC.

### 3.2. Spatiotemporal Dynamics and Trends of Alpine Grassland CGI

#### 3.2.1. CGI Spatiotemporal Distribution

**Figure 4:**
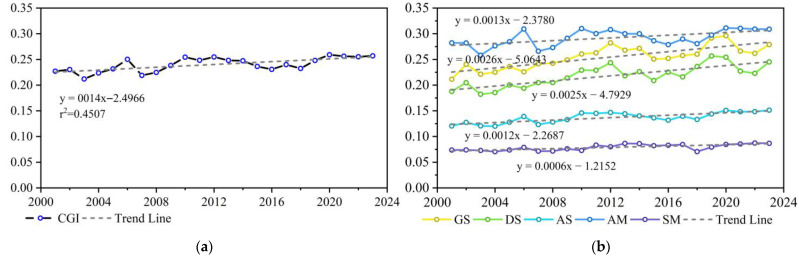
Interannual variation in the CGI of alpine grasslands in Qinghai Province (2001–2023): (**a**) overall alpine grasslands; (**b**) five alpine grassland types.

From 2001 to 2023, the CGI of alpine grasslands in Qinghai Province exhibited a fluctuating upward trend (slope ≈ 0.0014 year^−1^, *p* < 0.05), which was broadly divided into three phases. The CGI fluctuated markedly from 2001 to 2010, reaching its lowest value of 0.2120 in 2003; it showed a continuous decline from 2011 to 2016, followed by a fluctuating increase and subsequent stabilization from 2017 to 2023, peaking at 0.2591 in 2020 (Figure 4a). The CGI values across different grassland types also fluctuated but gradually increased over the 23 years (Figure 4b). Among them, alpine meadows showed the most pronounced interannual fluctuations in CGI compared with other grassland types.

**Figure 5:**
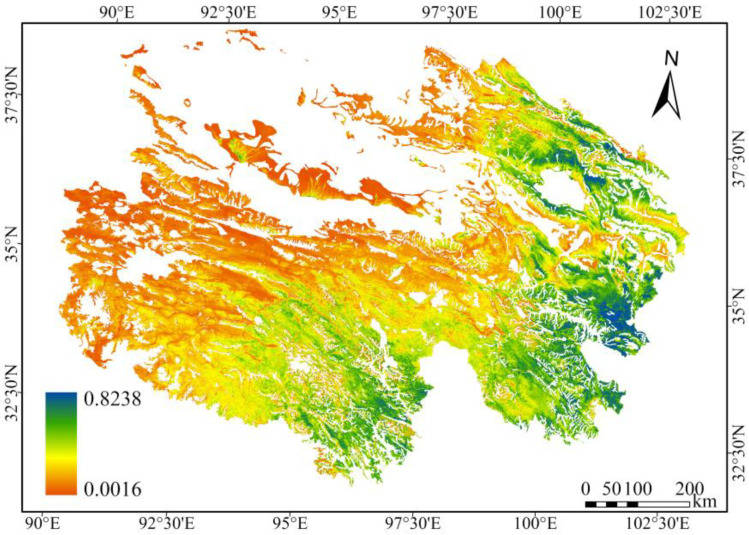
Spatial distribution of the CGI for alpine grasslands in Qinghai Province (2001–2023).

In this period, increases in the CGI were most pronounced in gramineous steppe and desert steppe, followed by alpine meadow and alpine steppe, whereas saline meadow showed minimal variation. The CGI slopes for each grassland type, in descending order, were as follows: gramineous steppe (0.0026 year^−1^) > desert steppe (0.0025 year^−1^) > alpine meadow (0.0013 year^−1^) > alpine steppe (0.0012 year^−1^) > saline meadow (0.0006 year^−1^). Significant differences existed in the CGI values among the grassland types, with the maximum values in descending order as follows: alpine meadow (0.3113, 2020) > gramineous steppe (0.2962, 2020) > desert steppe (0.2567, 2019) > alpine steppe (0.1512, 2023) > saline meadow (0.0873, 2022). Overall, the CGI of alpine grasslands in Qinghai Province exhibited a spatial distribution pattern, with higher values in the southeast and lower values in the northwest. High-value areas were mainly distributed in the alpine meadows of the eastern Yellow River headwaters within the Three-River-Source Region and around Qinghai Lake. Low-value areas were located mainly in the saline meadows of the central Qaidam Basin in the northwest and the alpine steppes of the Qiangtang Plateau (Figure 5).

**Figure 6:**
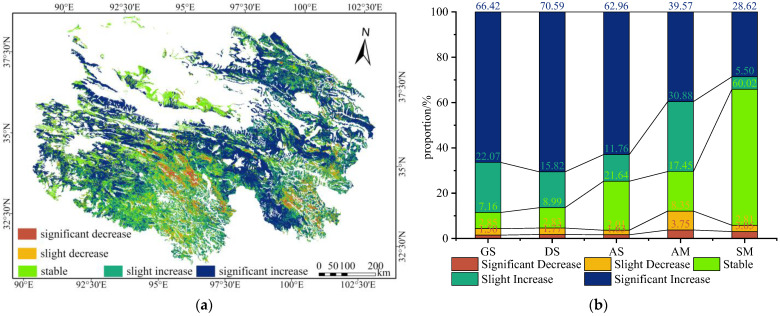
Spatiotemporal trends of the comprehensive growth index (CGI) for alpine grassland vegetation in Qinghai Province for 2001 to 2023. (**a**) Spatial distribution of CGI trends; (**b**) Classification of CGI trend grades.

Trend classification analysis (Figure 6a) further revealed that areas with significant and slight increases in CGI accounted for 46.57% and 24.67% of the total area, respectively, totaling 71.24%. CGI stable areas represented 19.41%, while significant and slight decreases made up only 9.35%. Spatially, increasing areas were concentrated in the alpine meadow zones of the Qilian Mountains and Yellow River headwaters, and in the western alpine meadow–alpine steppe zones of the Yangtze River headwaters. Stable areas were mainly located in the saline meadow zones of the Qaidam Basin, while decreasing areas were found in alpine meadow zones of the central Yangtze River and southeastern Yellow River headwaters.

The data in Figure 6b reveal distinct CGI change trends across the five alpine grassland types. The areas with a continuously increasing trend in CGI, ranked in descending order by proportion, were gramineous steppe (88.49%) > desert steppe (86.41%) > alpine steppe (74.72%) > alpine meadow (70.45%) > saline meadow (34.12%). Among these areas, the proportion of areas with significant increases in gramineous steppe, desert steppe, and alpine steppe ranged from 62.96% to 70.59%, while alpine meadow accounted for only 39.57%. The saline meadow exhibited the highest proportion of CGI-stable areas (60.02%), whereas the other four grassland types showed only 7.16–21.64%. The areas showing significant or slight decreases in the CGI were relatively small, accounting for only 3.64% to 12.10% of the total area. Alpine meadow made up the greatest share of this category, at approximately 12.10%.

#### 3.2.2. CGI Spatiotemporal Variation

**Figure 7:**
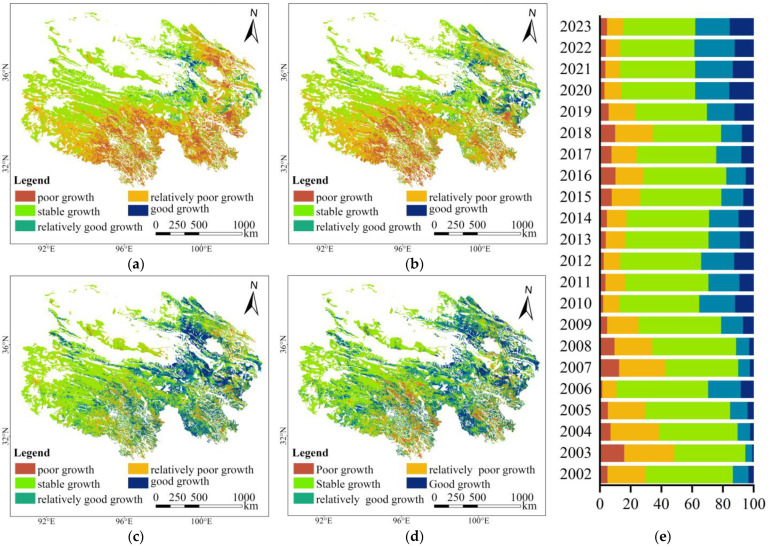
Classification of vegetation CGI differences (2002–2023 vs. 2001) for alpine grasslands in Qinghai Province. Panels (**a**–**d**) illustrate years with higher proportions of poor growth and relatively poor-growth areas ((**a**) 2003 and (**b**) 2007) and good growth and relatively good growth areas ((**c**) 2010 and (**d**) 2022). Panel (**e**) shows the area proportion of each category during 2002–2023 (Proportion, %).

**Figure 8:**
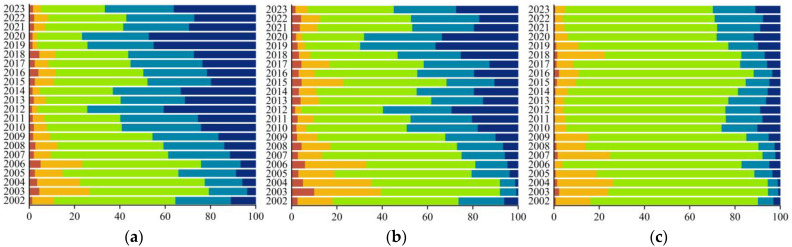

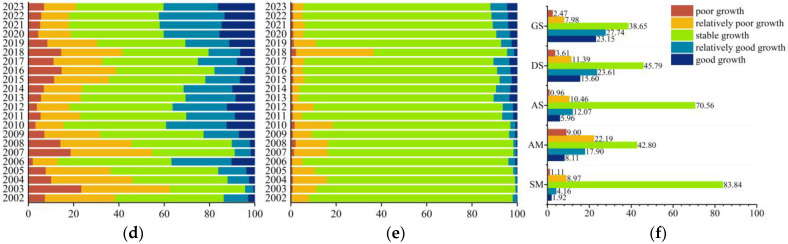
CGI difference classification of five types of alpine grasslands in Qinghai Province (2002–2023 vs. 2001). (**a**) gramineous steppe; (**b**) desert steppe; (**c**) alpine steppe; (**d**) alpine meadow; (**e**) saline meadow; (**f**) 2002–2023 mean proportion (Proportion, %).

Compared with the baseline year (2001), the overall growth status of alpine grasslands remained relatively stable from 2002 to 2023, accounting for approximately 44.44% to 59.48% of the total area each year (Figure 7). Areas with good and relatively good growth increased annually, from 13.33% in 2002 to 37.77% in 2023, whereas areas with poor and relatively poor growth declined from 29.72% in 2002 to 15.38% in 2023. Spatially, areas with stable growth were distributed mainly in the alpine steppe and saline meadow zones of northwestern Qinghai. Areas with good and relatively good growth were mainly located in alpine meadow, alpine steppe, gramineous steppe, and desert steppe zones of the Qinghai Lake Basin and its southern region. Areas with poor and relatively poor growth were primarily distributed in the alpine meadow zones of the southern Qilian Mountains and the Three-River Source Region. Compared with 2001, the proportion of areas with stable growth was the highest across all grassland types, averaging 38.65–83.84% annually (Figure 8). The proportion of areas with good and relatively good growth varied significantly among the grassland types in the following descending order: gramineous steppe (50.89%) > desert steppe (39.21%) > alpine meadow (26.01%) > alpine steppe (18.03%) > saline meadow (6.08%). Areas with poor and relatively poor growth accounted for a small proportion, particularly in alpine meadow zones (31.19%) and alpine steppe zones (11.42%). Overall, areas with stable growth across all five alpine grassland types decreased annually, whereas those with good and relatively good growth increased accordingly. Among these, the most pronounced growth improvement occurred in the gramineous steppe and desert steppe, whereas approximately one-quarter of the alpine meadow areas continued to decline.

### 3.3. Driving Factors of Alpine Grassland CGI

#### 3.3.1. Machine Learning Models Construction

CGI prediction models for different grassland types were constructed using RF, XGBoost, and LightGBM, with the mean CGI values from 2001 to 2023 used as the dependent variable and the mean values of various environmental factors used as independent variables. The data in Table 10 demonstrate that all three models achieved satisfactory CGI prediction performance for grassland vegetation. Among them, XGBoost performed best, followed by LightGBM. Both models yielded R^2^ values on the testing set ranging from 0.9239 to 0.9427 (excluding the saline meadow), with MAE and RMSE values between 0.0163 and 0.0410. The RF model exhibited relatively poor performance, with R^2^ values on the testing set ranging from 0.8356 to 0.9157 and MAE and RMSE values ranging from 0.0178 to 0.0553. The applicability of the three models in terms of CGI prediction across different grassland types also varied, with RF performing the worst and the other two models showing comparable performance. Desert steppe exhibited the highest prediction accuracy, followed by the alpine steppe, gramineous steppe, and alpine meadow. Both XGBoost and LightGBM achieved R^2^ values on the testing set exceeding 0.927, with the MAEs and RMSEs both being less than 0.041. The saline meadow showed comparatively lower prediction accuracy, with the testing set R^2^ being approximately 0.071 lower on average than those of the other grassland types, while the MAE values and RMSE values were about 0.008 and 0.004 lower, respectively. Overall, XGBoost demonstrated the highest predictive accuracy for CGI across all grassland types. Accordingly, XGBoost was adopted for subsequent CGI prediction under the comprehensive evaluation framework described in Section 2.3.5.

#### 3.3.2. Key Environmental Factors Identification by SHAP

On the basis of XGBoost, CGI prediction models were constructed for five alpine grassland types using 15 climatic, topographic, soil, and anthropogenic activity factors. The mean absolute SHAP value of each environmental factor was calculated to determine its relative contribution to the CGI. The six factors with the highest contribution values were selected as key factors influencing the CGI for each grassland type.

**Figure 9:**
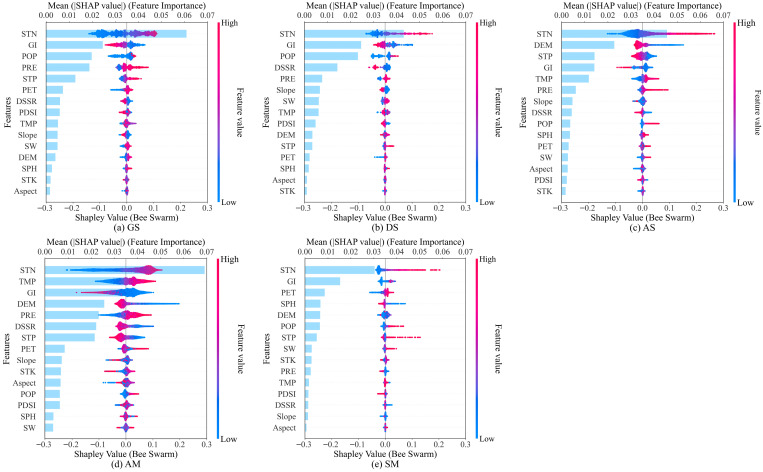
Feature importance and influence distribution based on the SHAP model. The vertical axis ranks features according to their mean absolute SHAP values, with importance decreasing from top to bottom. The horizontal axis represents SHAP values, where positive values indicate positive contributions and negative values indicate negative contributions. Each point represents a sample, and the color denotes feature magnitude (red = higher values, blue = lower values).

The factors influencing CGI across different grassland types exhibit both commonalities and distinct differences (Figure 9). Among the soil factors, STN was the most significant determinant of CGI across all grassland types. A higher STN content contributed positively to the CGI, with the following order of importance: gramineous steppe (33.58%) > saline meadow (32.20%) > alpine steppe (31.74%) > desert steppe (28.75%) > alpine meadow (24.78%). STP positively influenced the CGI in the gramineous steppe but negatively affected it in the alpine steppe. SW generally promoted the CGI in the desert steppe but showed negative effects in some localized regions. Among anthropogenic activity factors, GI ranked as the second most important influencing factor for the CGI across all grassland types, with the following order of importance: desert steppe (16.39%) > saline meadow (16.25%) > gramineous steppe (13.55%) > alpine meadow (12.13%) > alpine steppe (9.86%). A higher GI exerted a more substantial inhibitory effect on the CGI in the gramineous steppe, the desert steppe, the alpine steppe, and the alpine meadow. Moreover, in the saline meadow, the GI had both promotional and inhibitory effects on the CGI. Additionally, POP had a complex bidirectional effect on the CGI in the desert steppe and the gramineous steppe but contributed positively to the CGI in the saline meadow. Among the climatic factors, PRE and TMP were the primary shared influencing factors for the CGI across all grassland types except for the saline meadow. Increased PRE significantly promoted CGI, with the following order of importance: gramineous steppe (10.37%) > alpine meadow (8.33%) > desert steppe (5.13%) > alpine steppe (4.28%). TMP was among the most critical factors for the alpine meadow and had a significant positive influence on the CGI. Its importance ranking was alpine meadow (12.22%) > alpine steppe (8.21%) > desert steppe (4.10%) > gramineous steppe (2.84%). Other climatic factors exerted relatively weaker influences. Among the topographic factors, an increased DEM had a significant negative effect on the CGI in the alpine steppe and the alpine meadow but showed a nonlinear relationship with the CGI in the saline meadow.

#### 3.3.3. Dependence Between Key Driving Factors and CGI

**Figure 10:**
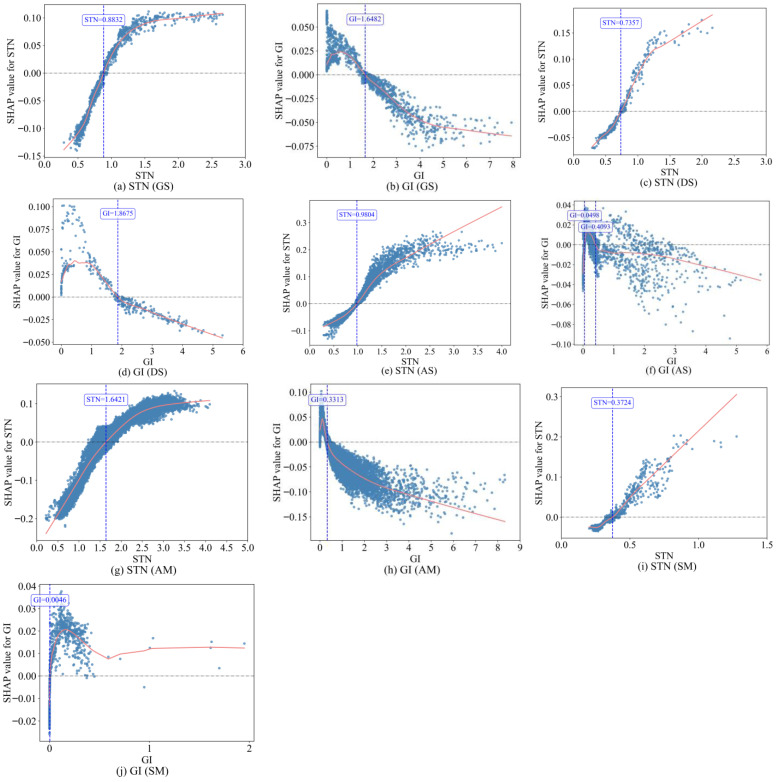
SHAP dependence plots. The horizontal axis represents the values of the influencing factors, and the vertical axis shows the corresponding SHAP values. The unit of STN is g·kg^−1^, and that of GI is SU·ha^−1^. Each dot represents an individual sample (pixel), and the solid red line indicates the smoothed dependencIe of SHAP values on the corresponding variable.

The common influencing factors STN and GI, shared across the five grassland types, were further analyzed using SHAP dependence plots (Figure 10) to explore their nonlinear relationships with the predicted CGI values. The results reveal that these two factors exert both common and distinct influences on the CGI among different grassland types. STN was the most significant influencing factor for CGI in alpine grasslands. Its response pattern, as indicated by the SHAP values across all five grassland types, was similar and significantly positively correlated, with the saline meadow exhibiting a clear linear positive relationship. When SHAP > 0, STN promoted the CGI, with distinct threshold values: alpine meadow (1.6421) > alpine steppe (0.9804) > gramineous steppe (0.8832) > desert steppe (0.7357) > saline meadow (0.3724). GI exhibited a similar influence trend on CGI across the five grassland types, but the relationship was relatively more complex. At lower GI values, it was positively correlated with the SHAP values for all grassland types, indicating that within a specific range, increasing the GI promoted the CGI. The optimal GI values (corresponding to the maximum SHAP values) for each grassland type, from highest to lowest, were as follows: gramineous steppe (0.5439) > desert steppe (0.4449) > saline meadow (0.1611) > alpine steppe (0.1391) > alpine meadow (0.1025). The GI and SHAP values subsequently exhibited a negative correlation (except for the saline meadow), indicating that the positive effect of increasing the GI on the CGI weakened. When SHAP < 0, the GI had an inhibitory effect on the CGI. The GI thresholds, ordered from largest to smallest, were desert steppe (1.8675) > gramineous steppe (1.6482) > alpine steppe (0.4093) > alpine meadow (0.3313). Notably, the impact of the GI on the CGI in the alpine steppe exhibited variability, revealing multiple thresholds (0.0498 and 0.4093). The effect of the GI on the CGI in the saline meadow differed markedly, clustering between 0.0046 and 0.5, where SHAP > 0. When the GI values exceeded 0.5, the SHAP values varied minimally with increasing GI, indicating that beyond a certain grazing intensity, the positive impact on the CGI became negligible and relatively stable.

## 4. Discussion

### 4.1. The Potential of the CGI Model in Alpine Grassland Growth Monitoring

The comprehensive vegetation growth model integrates multiple agronomic parameters, such as LAI, AGB, chlorophyll, nitrogen, and plant water content, thereby improving its applicability to crop growth monitoring [56,57]. Studies have shown that a CGI constructed from LAI and relative chlorophyll content (RCC) outperforms the single-index models for cotton growth monitoring in Shandong Province, China [58]. However, grasslands are much more complex than crops in composition, physiology, and phenology [59], and relying on single indices limits monitoring effectiveness [20]. To overcome these limitations, five vegetation indices representing key biophysical parameters were selected to construct a CGI model specific to alpine grasslands.

From 2001 to 2023, the interannual weight patterns of the five vegetation indices in the CGI model remained stable, demonstrating robustness (Figure 3). EVI and kNDVI received higher weights than MSAVI, GNDVI, and CVI, highlighting sensitivity to biomass, cover, and LAI, while also capturing chlorophyll, nitrogen, and soil background effects. This balance reflects the rationality of the model’s ecological representation. Although the CGI showed just average accuracy in FVC inversion (R^2^ ≈ 0.25), it outperformed the individual indices such as kNDVI. This indicates that the CGI provides a relatively more robust representation of alpine grassland growth by integrating complementary structural and biochemical information, particularly under heterogeneous environmental conditions.

### 4.2. The Spatiotemporal Variation in the Alpine Grassland Growth

The spatial variation in the CGI from 2001 to 2023 showed a general pattern of higher values in the southeast and lower in the northwest [26]. Temporally, the CGI showed significant interannual fluctuations during 2001–2023, and the minimum value of 0.2120 appeared in 2003, owing to the severe drought across western China during that period, which exerted detrimental effects on grassland growth [40]. Although grazing exclusion and restoration programs, such as the Returning Grazing Lands to Grasslands Project (GBP), have been implemented in Qinghai since the early 2000s [60,61], vegetation recovery exhibited a delayed response. Despite the implementation of Phase I of the Grassland Ecological Compensation Policy (GECP) during 2011–2015, the CGI continued to decline because of frequent droughts and initial policy adjustments [40,61,62]. Since 2017, CGI has exhibited a fluctuating upward trend and then leveled off, reaching a peak value of 0.2591 in 2020. This restoration aligned with the strengthened implementation of GECP Phase II (2016–2020) and Phase III (2021–2025), which featured expanded coverage and higher grazing-ban subsidy standards. These results suggest the combined influence of policy reinforcement and concurrent climatic conditions on alpine grassland growth [60,61].

Among the five grassland types, the gramineous steppe and desert steppe exhibited the greatest improvement, with over 39% of the area achieving good and relatively good growth and more than 85% showing increasing trends. Additionally, desert steppe is spatially adjacent to gramineous steppe, thus the improvements may stem from similar climatic, soil, and policy conditions. Alpine meadow and alpine steppe are the dominant vegetation types in Qinghai’s alpine grasslands, together covering 89.77% of the area and supporting regional livestock production. From 2001 to 2023, the proportions of areas with good and relatively good growth for the two types declined markedly to only 26.01% and 18.03%, and the proportions of areas with increasing growth trends also fell to 70.45% and 74.72%, respectively. Alpine meadows exhibited the highest CGI values but also showed strong interannual fluctuations and localized degradation. Areas characterized by good and continuously increasing growth were mainly distributed in the southern Qilian Mountains, the Qinghai Lake Basin, and the Yellow River headwaters, whereas continuous decreases occurred in parts of the Three-River Source Region, especially in the Yangtze headwaters [63]. Alpine steppes maintained relatively low but stable CGI values. The proportion of areas with stable growth reached 70.56%, and the proportion of areas with increasing trends was also higher than that of alpine meadows. Our results indicate that alpine meadows possess high ecological resilience but also exhibit greater sensitivity to external disturbances, such as overgrazing [64]. Although the simpler ecological structure may increase the degradation risk, alpine steppes demonstrate greater growth stability due to species adaptiveness to extreme alpine environments [65]. The saline meadow presented the lowest CGI values and minimal fluctuations, with 83.84% of the area in stable growth. Only 6.08% of the area achieved good and relatively good growth and 34.12% showed increasing trends. Constrained by saline soils and arid climatic conditions, vegetation growth remains poor and is difficult to improve substantially [66].

### 4.3. The Heterogeneity of Environmental Factors Affecting the Alpine Grassland Growth

Integrated with climatic, topographic, soil, and anthropogenic variables, this study evaluated the environmental responses of CGI across five alpine grassland types in Qinghai Province [25,26]. The XGBoost model coupled with SHAP exhibited excellent predictive performance for CGI and further revealed the complex environmental impacts on different grassland types, implying strong potential for ensemble learning in grassland ecological research [32].

Although significant variations exist in the primary drivers of vegetation growth across different grassland types in Qinghai Province, soil total nitrogen (STN) is the most critical common factor influencing CGI. This result is inconsistent with previous studies that highlighted climatic and anthropogenic factors [67]. STN directly regulates biomass accumulation and primary productivity and thus may exert more consistent control over CGI than short-term climate fluctuations [68,69,70]. The strong explanatory power of STN underscores the importance of improving soil nutrient conditions in alpine grassland management. Grazing intensity (GI) served as the second common driver; higher GI negatively affected vegetation growth in four types except saline meadow. The impacts on the growth of saline meadow were nonlinear, indicating that moderate grazing increases vegetation growth and the excessive grazing accelerates community degradation [2]. Our research revealed that the optimal GI threshold varied widely across the different types with a range of 0.1025–0.5439. GI has a stronger effect on desert steppe and saline meadow than on gramineous steppe and alpine meadow, indicating greater influence in arid than in humid grasslands [71]. Population (POP) has a greater effect on smaller-area grassland types: gramineous steppe, desert steppe, and saline meadow, suggesting that the three types are more sensitive to human disturbance than the widespread alpine meadow and alpine steppe. The results highlight the need for vegetation-type-specific grazing management and disturbance control strategies in terms of regional ecological carrying capacity, demonstrated by the empirical evidence from the Yellow River Source Region [72].

Compared with soil and anthropogenic factors, climatic factors play a less important role in influencing alpine grassland growth, which exert greater effects on the alpine meadow and the alpine steppe than the other three grassland types. PRE was the third common driver and generally promoted grassland growth (except for the saline meadow). TMP served as the second driver for the alpine meadow, implying a key determinant for the growth of alpine grasslands. The result agreed with the finding that reported temperature better explained vegetation dynamics on the Qinghai–Tibetan Plateau (QTP) [73], but differed from the research that emphasized the precipitation as the dominant factor for the grassland growth [74]. Zha et al. (2022) further revealed a geographic shift from PRE- to TMP-dominated regimes along the gradient from arid steppes to humid meadows [75]. These discrepancies may be related to grassland-type specificity and spatiotemporal scale and need further investigation. DEM had significant influence on the alpine steppe and alpine meadow, indicating the poor growth in high-elevation regions due to nutrient-insufficient soils [76]. Prior studies found that PRE dominated the growth below 3400 m, while TMP plays a key role above 3400 m in elevation [77]. Therefore, future work should be performed to elucidate the interaction among topography, climate, and soil.

## 5. Conclusions

The study proposed an integrated method for assessing the long-term vegetation dynamics and their ecological drivers in the alpine grassland of Qinghai Province. The constructed CGI model effectively represented the multi-dimensional vegetation parameters and depicted the growth difference across five major grassland types, reflecting diverse ecological sensitivities and adaptive capacities. Soil total nitrogen (STN) served as the dominant driver for the growth across all grasslands, while grazing intensity (GI) and key climatic factors (temperature and precipitation) exerted nonlinear and vegetation-type-specific effects. These findings highlight the importance of enhancing soil nutrient availability, implementing adaptive grazing strategies, and promoting climate-resilient management practices.

## Figures and Tables

**Table 1:** MOD13Q1 and MOD09A parameters.

Data Type	Band/Index	Wavelength (nm)	Spatial/Temporal Resolution
MOD13Q1	Red	620–670	250 m/16 d
	NIR	841–876	
	EVI	/	
MOD09A1	Red	620–670	500 m/8 d
	NIR	841–876	
	Blue	459–479	
	Green	545–565	

**Table 2:** Numbers of AGB and FVC quadrats collected.

Type	Year	Number_A	Number_B
AGB	2005	747	202
	2006	748	112
FVC	2015	342	296
	2016	561	484

Note: Number_A—quadrats obtained from dataset; Number_B—quadrats retained after data processing.

**Table 3:** Environmental factors used in this study.

Environmental Factor		Resolution	Dataset
Climate	Precipitation/PRE	1000 m	China Precipitation, Mean Temperature, and Potential Evapotranspiration Dataset (monthly, 1901–2024), National Tibetan Plateau Data Center (TPDC), Third Pole Environment Data Center (https://data.tpdc.ac.cn).
	Temperature/TMP		
	Potential evapotranspiration/PET		
	Downward surface shortwave radiation/DSSR Palmer drought severity index/PDSI	4500 m	TerraClimate Dataset (monthly, 1958–2024), GEE (https://developers.google.com/earth-engine/datasets/catalog/IDAHO_EPSCOR_TERRACLIMATE, accessed on 26 July 2025).
Topography	DEM	30 m	Copernicus Global 30 m Digital Elevation Model (GLO-30 DEM), released by the European Space Agency (ESA), GEE (https://developers.google.com/earth-engine/datasets/catalog/COPERNICUS_DEM_GLO30, accessed on 26 July 2025).
	Slope		
	Aspect		
Soil	Soil total nitrogen/STN	250 m	Basic Soil Property Dataset of High-Resolution China Soil Information Grids (2010–2018), National Tibetan Plateau Data Center (TPDC), Third Pole Environment Data Center (https://data.tpdc.ac.cn).
	Soil total potassium/STK		
	Soil total phosphorus/STP		
	Soil PH/SPH		
	Soil water/SW	0.1°	ERA5-Land Monthly Aggregated Dataset (Since 1950), GEE (https://developers.google.com/earth-engine/datasets/catalog/ECMWF_ERA5_LAND_MONTHLY_AGGR, accessed on 26 July 2025).
Anthropogenic activity	Grazing intensity/GI	250 m	Long-term High-resolution Grazing Intensity Dataset (yearly, 2001–2024), National Science and Technology Infrastructure of China (https://www.nesdc.org.cn/).
	Population/POP	1000 m	LandScan Global 1 km Population Dataset, ORNL, U.S. Department of Energy (yearly, 2000–2023), GEE (https://developers.google.com/earth-engine/datasets/catalog/projects_sat-io_open-datasets_ORNL_LANDSCAN_GLOBAL?hl=zh-cn, accessed on 26 July 2025).

**Table 4:** Selected vegetation indices.

Index	Formula	Application
Kernel normalized difference vegetation index (kNDVI)	$\mathrm{kNDVI}=\tan h\left(\frac{{\left(\rho_{\mathrm{NIR}}-\rho_{\mathrm{Red}}\right)}^{2}}{{\left(2\mathrm{sigma}\right)}^{2}}\right)$ $\;\mathrm{sigma}=0.5\left(\rho_{\mathrm{NIR}}+\rho_{\mathrm{Red}}\right)$	Sensitive to canopy structural and biochemical properties (e.g., LAI and chlorophyll) and alleviates NDVI saturation [46].
Enhanced vegetation index (EVI)	$\mathrm{EVI}=2.5\times \frac{\left(\rho_{\mathrm{NIR}}-\rho_{\mathrm{Red}}\right)}{{(\rho }_{\mathrm{NIR}}+6\rho_{\mathrm{Red}}-7.5\rho_{\mathrm{Blue}}+1)}$	Enhances sensitivity under high-biomass conditions and reduces atmospheric effects [47].
Ratio vegetation index (RVI)	$\mathrm{RVI}=\frac{\rho_{\mathrm{NIR}}}{\rho_{\mathrm{Red}}}$	Effective for estimating shrub aboveground biomass in arid and semi-arid regions [11].
Modified soil-adjusted vegetation index (MSAVI)	$\mathrm{MSAVI}=\frac{2\rho_{\mathrm{NIR}}+1-\sqrt{{\left({2\rho }_{\mathrm{NIR}}+1\right)}^{2}-8\left(\rho_{\mathrm{NIR}}-\rho_{\mathrm{Red}}\right)}\;}{2}$	Minimizes bare soil influence and highlights sparse vegetation [12].
Green chlorophyll index (GCI)	$\mathrm{GCI}=\frac{\rho_{\mathrm{NIR}}}{\rho_{\mathrm{Green}}}-1$	Sensitive to chlorophyll content [13].
Chlorophyll vegetation index (CVI)	$\mathrm{CVI}=\frac{\rho_{\mathrm{NIR}}\times \rho_{\mathrm{Red}}}{\rho_{\mathrm{Green}}^{2}}$	Indicates chlorophyll content and canopy biochemical status [14].
Green normalized difference vegetation index (GNDVI)	$\mathrm{GNDVI}=\frac{\rho_{\mathrm{NIR}}-\rho_{\mathrm{Green}}}{\rho_{\mathrm{NIR}}+\rho_{\mathrm{Green}}}$	Reflects chlorophyll content and is strongly associated with FVC in alpine meadows [15,16].
Normalized difference vegetation index green-blue (NDVI_green-blue_)	${\mathrm{NDVI}}_{\mathrm{green}-\mathrm{blue}}=\frac{\rho_{\mathrm{Green}}-\rho_{\mathrm{Blue}}}{\rho_{\mathrm{Green}}+\rho_{\mathrm{Blue}}}$	Sensitive to leaf nitrogen content based on green–blue spectral information [17].

Note: ρ_Blue_, ρ_Green_, ρ_Red_, and ρ_NIR_ denote the surface reflectances of the blue, green, red, and near-infrared bands of the MODIS imagery, respectively.

**Table 5:** CGI difference classification (2002–2023 vs. 2001).

CGI Difference	Level
<−0.0503	poor growth
−0.0503–−0.0082	relatively poor growth
−0.0082–0.0339	stable growth
0.0339–0.0760	relatively good growth
>0.0760	good growth

**Table 6:** Grading of vegetation growth trend (Sen–MK Method).

β	Z_c_	Level
β < −0.0005	Z_c_ < −1.96	significant decrease
β < −0.0005	−1.96 ≤ Z_c_ < 1.96	slight decrease
−0.0005 ≤ β < 0.0005	−1.96 ≤ Z_c_ < 1.96	stable
β ≥ 0.0005	−1.96 ≤ Z_c_ < 1.96	slight increase
β ≥ 0.0005	Z_c_ ≥ 1.96	significant increase

Note: β denotes the estimator of Sen’s slope, and Z_c_ represents the Mann–Kendall test statistic. A time series is considered significant at the 95% confidence level when |Z_c_| > 1.96.

**Table 7:** Optimization strategies for machine learning models.

Model	Parameters	Scale
RF	max_depth	(5, 8)
	min_samples_split	(2, 10)
	min_samples_leaf	(5, 15)
XGBoost	learning_rate	(0.01, 0.1)
	max_depth	(5, 8)
	subsample	(0.5, 0.9)
	min_child_weight	(10, 20)
	colsample_bytree	(0.2, 0.9)
LightGBM	learning_rate	(0.01, 0.1)
	max_depth	(5, 8)
	num_leaves	(10, 30)
	feature_fraction	(0.2, 0.9)

**Table 8:** Total score of vegetation index combinations.

Vegetation Index Combination	Total Score
kNDVI, EVI, MSAVI, GNDVI, CVI	14.8057
kNDVI, MSAVI, GNDVI, CVI, RVI, GCI	14.5179
kNDVI, MSAVI, GNDVI, NDVI_green-blue_, CVI, RVI, GCI	14.4377
EVI, MSAVI, GNDVI, CVI, RVI, GCI	14.3582
MSAVI, GNDVI, CVI, RVI, GCI	14.2688

Note: The top 5 combinations are listed.

**Table 9:** Accuracy validation based on 2022 FVC data.

Year	Types	R^2^_CGI_	R^2^_kNDVI_	RMSE_CGI_	RMSE_kNDVI_
2022	FVC	0.2475	0.2286	19.7692	20.0160

**Table 10:** Accuracy achieved by RF, XGBoost and LightGBM.

Grassland Type	Model	Training Set			Testing Set		
		R^2^	MAE	RMSE	R^2^	MAE	RMSE
Gramineous Steppe	RF	0.9482	0.0223	0.0312	0.9082	0.2907	0.0417
	XGBoost	0.9767	0.0156	0.0209	0.9274	0.0261	0.0371
	LightGBM	0.9865	0.0118	0.0160	0.9275	0.0261	0.0371
Desert Steppe	RF	0.9577	0.0161	0.0239	0.9157	0.0236	0.0379
	XGBoost	0.9736	0.0136	0.0188	0.9427	0.0217	0.0313
	LightGBM	0.9609	0.0162	0.0229	0.9318	0.0233	0.0341
Alpine Steppe	RF	0.9129	0.0222	0.0308	0.8952	0.0237	0.0339
	XGBoost	0.9647	0.0143	0.0196	0.9358	0.0184	0.0265
	LightGBM	0.9597	0.0149	0.0209	0.9271	0.0195	0.0283
Alpine Meadow	RF	0.8727	0.0394	0.0529	0.8617	0.0411	0.0553
	XGBoost	0.9787	0.0164	0.0216	0.9306	0.0289	0.0392
	LightGBM	0.9530	0.0240	0.0321	0.9239	0.0303	0.041
Saline Meadow	RF	0.9036	0.0131	0.0242	0.8356	0.0178	0.0326
	XGBoost	0.9775	0.0076	0.0117	0.8629	0.0163	0.0297
	LightGBM	0.9984	0.0022	0.0031	0.857	0.0165	0.0303

## Data Availability

The data that support the findings of this study are available on request from the corresponding author.

## References

[1] Yan L., Li Y., Wang L., Zhang X., Wang J., Wu H., Yan Z., Zhang K., Kang X. (2020). Grazing significantly increases root shoot ratio but decreases soil organic carbon in Qinghai-Tibetan Plateau grasslands: A hierarchical meta-analysis. Land Degrad. Dev..

[2] Zhang Z., Zhao Y., Lin H., Li Y., Fu J., Wang Y., Sun J., Zhao Y. (2023). Comprehensive analysis of grazing intensity impacts alpine grasslands across the Qinghai–Tibetan Plateau: A meta-analysis. Front. Plant Sci..

[3] Zhang Z., Liu Y., Sun J., Wu G.-L. (2021). Suitable duration of grazing exclusion for restoration of a degraded alpine meadow on the eastern Qinghai–Tibetan Plateau. Catena.

[4] Chen J., Yan F., Lu Q. (2020). Spatiotemporal Variation of Vegetation on the Qinghai–Tibet Plateau and the Influence of Climatic Factors and Human Activities on Vegetation Trend (2000–2019). Remote Sens..

[5] Rao X., Li H., Zhang S., Luo M., Liu Z., Zhang J. (2021). Suitability analysis of remote sensing monitoring methods for grassland vegetation growth. Chin. J. Eco-Agric..

[6] Wang Y., Qin R., Cheng H., Liang T., Zhang K., Chai N., Gao J., Feng Q., Hou M., Liu J. (2022). Can machine learning algorithms successfully predict grassland aboveground biomass?. Remote Sens..

[7] Lin X., Chen J., Lou P., Yi S., Qin Y., You H., Han X. (2021). Improving the estimation of alpine grassland fractional vegetation cover using optimized algorithms and multi-dimensional features. Plant Methods.

[8] Pettorelli N., Vik J.O., Mysterud A., Gaillard J.-M., Tucker C.J., Stenseth N.C. (2005). Using the satellite-derived NDVI to assess ecological responses to environmental change. Trends Ecol. Evol..

[9] Feng X., Tian J., Wu J., Wu G., Ren Y., He C., Bao W., Yu T. (2025). Exploring the spatio-temporal distribution characteristics and the impacts of climate change and human activities on global grassland based on kNDVI. Environ. Res..

[10] Ma C., Xie Y., Duan H., Wang X., Bie Q., Guo Z., He L., Qin W. (2022). Spatial quantification method of grassland utilization intensity on the Qinghai–Tibetan Plateau: A case study on the Selinco Basin. J. Environ. Manag..

[11] Xu M., Cao C., Tong Q., Li Z., Zhang H., He Q., Gao M., Zhao J., Zheng S., Chen W. (2010). Remote sensing-based shrub above-ground biomass and carbon storage mapping in Mu Us Desert, China. Sci. China Technol. Sci..

[12] Qi J., Huete A.R., Kerr Y.H., Sorooshian S. (1994). A modified soil adjusted vegetation index. Remote Sens. Environ..

[13] Gitelson A.A., Gritz Y., Merzlyak M.N. (2003). Relationships between leaf chlorophyll content and spectral reflectance and algorithms for non-destructive chlorophyll assessment in higher plant leaves. J. Plant Physiol..

[14] Vincini M., Frazzi E. (2011). Comparing narrow- and broad-band vegetation indices to estimate leaf chlorophyll content in planophile crop canopies. Precis. Agric..

[15] Gao S., Yan K., Liu J., Pu J., Zou D., Qi J., Mu X., Yan G. (2024). Assessment of remote-sensed vegetation indices for estimating forest chlorophyll concentration. Ecol. Indic..

[16] Du K., Shao Y., Yao N., Yu H., Ma S., Mao X., Wang L., Wang J. (2025). Alpine meadow fractional vegetation cover estimation using UAV-aided Sentinel-2 imagery. Sensors.

[17] Fan L., Zhao J., Xu X., Liang D., Yang G., Feng H., Yang H., Wang Y., Chen G., Wei P. (2019). Hyperspectral-based estimation of leaf nitrogen content in corn using optimal selection of multiple spectral variables. Sensors.

[18] Fan Y., Feng H., Liu Y., Feng H., Yue J., Jin X., Chen R., Bian M., Ma Y., Yang G. (2024). Transferability of models for predicting potato plant nitrogen content from remote sensing data and environmental variables across years and regions. Eur. J. Agron..

[19] Zhao X., Na X. (2022). Study on remote sensing monitoring of crop growth based on comprehensive index: A case study of the Songnen Plain. Geogr. Geo-Inf. Sci..

[20] Li Q., Cheng J., Yan J., Zhang G., Ling H. (2025). Comparison of satellite-derived vegetation indices for assessing vegetation dynamics in Central Asia. Water.

[21] Chen B., Zhang X., Tao J., Wu J., Wang J., Shi P., Zhang Y., Yu C. (2014). The impact of climate change and anthropogenic activities on alpine grassland over the Qinghai–Tibet Plateau. Agric. For. Meteorol..

[22] Yao T., Xue Y., Chen D., Chen F., Thompson L., Cui P., Koike T., Lau W.K.M., Lettenmaier D., Mosbrugger V. (2019). Recent Third Pole’s rapid warming accompanies cryospheric melt and water cycle intensification and interactions between monsoon and environment. Bull. Am. Meteorol. Soc..

[23] Liu C., Li W., Wang W., Zhou H., Liang T., Hou F., Xu J., Xue P. (2021). Quantitative spatial analysis of vegetation dynamics and potential driving factors in a typical alpine region on the northeastern Tibetan Plateau using Google Earth Engine. Catena.

[24] Xie H.H., Wu Q.G., Hu J.Y., Yu L.F., Bie P.F., Wang H., Deng D.Z. (2018). Changes in soil physical and chemical properties during the process of alpine meadow degradation along the eastern Qinghai–Tibet Plateau. Eurasian Soil Sci..

[25] Wu X., Liu H., Li X., Ciais P., Babst F., Guo W., Zhang C., Magliulo V., Pavelka M., Liu S. (2018). Differentiating drought legacy effects on vegetation growth over the temperate Northern Hemisphere. Glob. Change Biol..

[26] Wang H., Zhan J., Wang C., Liu W., Yang Z., Liu H., Bai C. (2022). Greening or browning? Macro-variation and drivers of different vegetation types on the Qinghai–Tibetan Plateau from 2000 to 2021. Front. Plant Sci..

[27] Xia Z., Liao K., Guo L., Wang B., Huang H., Chen X., Fang X., Zu K., Luo Z., Shen F. (2025). Determining dominant factors of vegetation change with machine learning and multisource data in the Ganjiang River Basin, China. Land.

[28] Kolluru V., John R., Chen J., Xiao J., Amirkhiz R.G., Giannico V., Kussainova M. (2022). Optimal ranges of social–environmental drivers and their impacts on vegetation dynamics in Kazakhstan. Sci. Total Environ..

[29] Qiao Y., Chen H., Jiang Y. (2020). Quantifying the impacts of lithology on vegetation restoration using a random forest model in a karst trough valley, China. Ecol. Eng..

[30] Gao G., Feng Q., Xu E., Hao Y., Wang R., Jing W., Ren X., Shi J., Wu B., Wen Y. (2025). Carbon dioxide and water exchanges of a Qinghai spruce forest ecosystem in the Qilian Mountains, Northwestern China. J. Hydrol..

[31] Zhang X., Wang G., Xue B., A Y. (2022). Changes in vegetation cover and its influencing factors in the Inner Mongolia reach of the Yellow River Basin from 2001 to 2018. Environ. Res..

[32] Ming L., Wang Y., Liu G., Meng L., Chen X. (2024). Analysis of vegetation dynamics from 2001 to 2020 in China’s Ganzhou rare earth mining area using time-series remote sensing and SHAP-enhanced machine learning. Ecol. Inform..

[33] Geng Y., Wang Z., Liang C., Fang J., Baumann F., Kühn P., Scholten T., He J.S. (2012). Effect of geographical range size on plant functional traits and the relationships between plant, soil and climate in Chinese grasslands. Glob. Ecol. Biogeogr..

[34] Simovic I., Ocokoljic M., Obratov-Petkovic D., Vilotic D. (2015). Genetic variability of bilaterally symmetrical fruits of Norway maple in function of species biodiversity conservation. Turk. J. Agric. For..

[35] Gao F., Anderson M.C., Zhang X., Yang Z., Alfieri J.G., Kustas W.P., Mueller R., Johnson D.M., Prueger J.H. (2017). Toward mapping crop progress at field scales through fusion of Landsat and MODIS imagery. Remote Sens. Environ..

[36] Hao A., Xue X., Peng F., You Q., Liao J., Duan H., Huang C., Dong S. (2020). Different vegetation and soil degradation characteristics of a typical grassland in the Qinghai–Tibet Plateau. Acta Ecol. Sin..

[37] Dong S., Shang Z., Gao J., Boone R. (2021). Enhancing sustainability of grassland ecosystems through ecological restoration and grazing management in an era of climate change on the Qinghai–Tibetan Plateau. Agric. Ecosyst. Environ..

[38] Wang S., Dai E., Jia L., Wang Y., Huang A., Liao L., Cai L., Fan D. (2023). Assessment of multiple factors and interactions affecting grassland degradation on the Tibetan Plateau. Ecol. Indic..

[39] Kuang X., Jiao J. (2016). Review on climate change on the Tibetan Plateau during the last half century. J. Geophys. Res. Atmos..

[40] Cao S., He Y., Zhang L., Chen Y., Yang W., Yao S., Sun Q. (2021). Spatiotemporal characteristics of drought and its impact on vegetation in Northwest China. Ecol. Indic..

[41] Huang X., Chen C., Yao B., Ma Z., Zhou H. (2021). Spatiotemporal dynamics of the carbon budget and the response to grazing in Qinghai grasslands. Front. Plant Sci..

[42] Zhang C., Brodylo D., Rahman M., Rahman M.A., Douglas T.A., Comas X. (2022). Using an object-based machine learning ensemble approach to upscale evapotranspiration measured from eddy covariance towers in a subtropical wetland. Sci. Total Environ..

[43] Xia J., Ma M., Liang T., Wu C., Yang Y., Zhang L., Zhang Y., Yuan W. (2018). Estimates of grassland biomass and turnover time on the Tibetan Plateau. Environ. Res. Lett..

[44] Liu J., Huang X., He X., Shen K. (2018). Estimation of grassland yield and carrying capacity in Qinghai Province based on MODIS data. Pratacult. Sci..

[45] Zhang X. (2007). Vegetation Map of the People’s Republic of China (1:1,000,000).

[46] Wang Q., Moreno-Martínez Á., Muñoz-Marí J., Campos-Taberner M., Camps-Valls G. (2023). Estimation of vegetation traits with kernel NDVI. ISPRS J. Photogramm. Remote Sens..

[47] Huete A., Didan K., Miura T., Rodriguez E.P., Gao X., Ferreira L.G. (2002). Overview of the radiometric and biophysical performance of the MODIS vegetation indices. Remote Sens. Environ..

[48] Chen Y., Qiu Y., Zhang Z., Zhang J., Chen C., Han J., Liu D. (2020). Estimating salt content of vegetated soil at different depths with Sentinel-2 data. PeerJ.

[49] Akaike H., Parzen E., Tanabe K., Kitagawa G. (1998). Information theory and an extension of the maximum likelihood principle. Selected Papers of Hirotugu Akaike.

[50] Cao Y., Qian D., Wang C. (2023). Quantitative traceability study on the water quality driving forces across cities in the Xiang River Basin. Water Supply.

[51] Yan M., Lu J., Ma Y., Ma C. (2025). Remote sensing evidence on marginality, fragmentation and spatiotemporal heterogeneity of vegetation evolution in the Yinshan Mountains (1984–2022). Ecol. Indic..

[52] Liaw A., Wiener M. (2002). Classification and regression by RandomForest. R News.

[53] Jia Z., Zhang Z., Cheng Y., Buhebaoyin, Borjigin S., Quan Z. (2024). Grassland biomass spatiotemporal patterns and response to climate change in eastern Inner Mongolia based on XGBoost estimates. Ecol. Indic..

[54] Ke G., Meng Q., Finley T., Wang T., Chen W., Ma W., Ye Q., Liu T.-Y. LightGBM: A highly efficient gradient boosting decision tree. Proceedings of the 31st International Conference on Neural Information Processing Systems.

[55] Li X., Chen J., Chen Z., Lan Y., Ling M., Huang Q., Li H., Han X., Yi S. (2024). Explainable machine learning-based fractional vegetation cover inversion and performance optimization: A case study of an alpine grassland on the Qinghai–Tibet Plateau. Ecol. Inform..

[56] Tang Y., Zhou Y., Cheng M., Sun C. (2023). Comprehensive growth index (CGI): A comprehensive indicator from UAV-observed data for winter wheat growth monitoring. Agronomy.

[57] Feng H., Tao H., Li Z., Yang G., Zhao C. (2022). Comparison of UAV RGB imagery and hyperspectral remote sensing data for monitoring winter wheat growth. Remote Sens..

[58] Gu H., Xue C., Wang G., Lan Y., Wang H., Song C. (2024). UAV-based multispectral inversion of integrated cotton growth. Agronomy.

[59] McGonigle T.P., Turner W.G. (2017). Grasslands and croplands have different microbial biomass carbon levels per unit of soil organic carbon. Agriculture.

[60] Dong S., Xu Y., Li S., Shen H., Yang M., Xiao J. (2024). Restoration actions associated with payment for ecosystem services promote economic returns of alpine grasslands in China. J. Clean. Prod..

[61] Yang F., Xu J., Zhao X., Wang X., Xiong Y. (2022). Assessment of the grassland ecological compensation policy in Qinghai, China. Agriculture.

[62] Liu M., Bai L., Khan H.S., Li H. (2023). Influence of grassland ecological compensation policy on herdsmen’s income and income gap. Agriculture.

[63] Shen X., An R., Feng L., Ye N., Zhu L., Li M. (2018). Vegetation changes in the Three-River Headwaters Region of the Tibetan Plateau. Ecol. Indic..

[64] Hao A., Duan H., Wang X., Zhao G., You Q., Peng F., Du H., Liu F., Li C., Lai C. (2021). Different responses of alpine meadow and alpine steppe to climatic and anthropogenic disturbance on the Qinghai–Tibetan Plateau. Glob. Ecol. Conserv..

[65] Tang L., Sherman R., Liu S., Liu Q., Wang X., Su X., Zhang Y., Li Y., Wu Y., Zhao H. (2015). Changes in vegetation composition and plant diversity with rangeland degradation in the alpine region of the Qinghai–Tibet Plateau. Rangel. J..

[66] Zhao Y., Chen H., Sun H., Yang F. (2024). Soil nutrients directly or indirectly affect desert ecosystem stability under drought stress in the Qaidam Basin. Plants.

[67] Sun L., Li H., Wang J., Chen Y., Xiong N., Wang Z., Wang J., Xu J. (2023). Impacts of climate change and human activities on NDVI on the Qinghai–Tibet Plateau. Remote Sens..

[68] Nie X., Xiong F., Yang L., Li C., Zhou G. (2017). Soil nitrogen storage, distribution, and controlling factors in shrublands of the northeastern Tibetan Plateau. Forests.

[69] Wang X., Wang R., Gao J. (2022). Precipitation and soil nutrients determine the spatial variability of grassland productivity at large scales in China. Front. Plant Sci..

[70] Kou D., Yang G., Li F., Feng X., Zhang D., Mao C., Zhang Q., Peng Y., Ji C., Zhu Q. (2020). Progressive nitrogen limitation across the Tibetan alpine permafrost region. Nat. Commun..

[71] Lv S., Huang J., Liu H., Ma S. (2024). Grazing effects on species diversity across scales are related to grassland types. BMC Plant Biol..

[72] Hu X., Wang Z., Zhang Y., Gong D., Liu L., Li K. (2025). Effectiveness of conservation measures based on assessment of grazing intensity in the Yellow River Source Region. Land.

[73] Li Z., Qu H., Li L., Zheng J., Wei D., Wang F. (2023). Effects of climate change on vegetation dynamics on the Qinghai–Tibet Plateau: A causality analysis using empirical dynamic modeling. Heliyon.

[74] Sun J., Qin X., Yang J. (2015). Responses of vegetation dynamics of different alpine grassland types to temperature and precipitation on the Tibetan Plateau. Environ. Monit. Assess..

[75] Zha X., Niu B., Li M., Duan C. (2022). Increasing impact of precipitation on alpine grassland productivity over the last two decades on the Tibetan Plateau. Remote Sens..

[76] Li C., de Jong R., Schmid B., Wulf H., Schaepman M.E. (2020). Changes in grassland cover and its spatial heterogeneity indicate degradation on the Qinghai–Tibetan Plateau. Ecol. Indic..

[77] Tang Z., Zhang Y., Lei M., Li Z., Zhao G., Chen Y., Zhu W. (2024). Climate-driven effects on NPP in Tibetan Plateau alpine grasslands diminish with increasing elevation. Remote Sens..

